# Diagnosis of Alzheimer’s Disease with [18F]PET in Mild and Asymptomatic Stages

**DOI:** 10.3233/BEN-2009-0228

**Published:** 2009-10-21

**Authors:** Alexander Drzezga

**Affiliations:** ^1^Harvard Medical SchoolMassachusetts General HospitalAthinoula A. Martinos Center for Biomedical ImagingCharlestownMAUSA; ^2^Department of Nuclear MedicineKlinikum rechts der IsarTechnische Universität MünchenMunichGermany

## Abstract

With longer life expectancy, dementia based on the age-related Alzheimers’ disease (AD) has turned into one of the most prevalent disorders of older age, representing a serious medical and socio-economic issue. There has been growing interest in early diagnosis of this disease, particularly regarding the initiation of new treatment strategies ahead of the onset of irreversible neuronal damage. It is accepted that the pathologic changes underlying AD appear in the brain years to decades before the symptomatic stages. Consequently, clinical measures of cognitive impairment, as used for definition of dementia, will not allow early diagnosis of AD-pathology in the mild or asymptomatic stages. Thus, a need for complementary sensitive biomarkers is apparent. Brain imaging markers are among the most promising candidates for this diagnostic challenge. Particularly, [18F]FDG PET as a marker of regional neuronal function has been demonstrated to represent a most sensitive and specific method for early identification of AD-pathology and thus for prediction of dementia of the Alzheimer type (DAT), even in the mild and asymptomatic stages. Currently, systematic data of comparable quality are hardly available for any other imaging procedure. The purpose of this article is to describe the typical findings of [18F]FDG PET in different stages of AD and to demonstrate its value for early and reliable diagnosis of Alzheimer's disease, particularly ahead of the stage of dementia of the Alzheimer’s type.

## 1. Introduction

Age represents the greatest known risk factor for dementia. The prevalence of the disease shows an almost exponential increase with age [10]. More than 20% of persons older than 80 years are affected by manifest dementia [9,36] and the risk doubles approximately every 5 years [2,119]. Most of these dementia cases of older age are based on the neurodegenerative disorder Alzheimer's disease (AD) [14]. With increasing lifespan, dementia turns into one of the most prevalent and debilitating disorders in today's societies, characterized by an insidious onset and a progressive impairment of cognitive functions, finally leading to an impairment in the capabilities of daily living [33,60]. Only in the United States up to 5 million individuals are estimated to suffer from Alzheimer's disease today [1]. AD already represents the fifth leading cause of death in Americans older than the age of 65 years. Regarding the fact that the elderly population (those aged 65 years or older) in the USA is expected to duplicate from approximately 35 million today to more than 70 million by 2030, the incidence of Alzheimer's disease is expected to approach nearly a million people per year, with a total estimated prevalence of 11 to 16 million persons by 2050 [87] These facts illustrate that this disorder will represent an enormous burden in the future, not only for the patients and their relatives but also regarding socio-economic and health care systems.

Today's diagnostic algorithms for the diagnostic verification of AD are still mainly directed towards the definition of manifest dementia based on clinical symptoms [60]. The diagnostic standards of the NINCDS-ADRDA rely on the exclusion of other causes for dementia, leaving AD as the end point of a long diagnostic road with neuropsychological examination as the

ISSN 0953-4180/09/$17.00 © 2009 – IOS Press and the authors. All rights reserved most important tool. These tools unfortunately have a rather low sensitivity and specificity with 80% and 70% respectively- these numbers might even be lower particularly when looking at the crucial early stages of AD [52]. The systematic algorithm of the clinical diagnosis of dementia is currently divided into two steps: 1.) Confirmation of the diagnosis "dementia" and 2.) Differential diagnosis of the cause underlying the dementia. Step one is usually carried out on the basis of an interview with the patient and usually also with at least one informant (e.g. spouse or children of the patient), as well as by clinical neuropsychological testing of the patient. The second step also includes laboratory tests and other technical examinations such as ECG and brain imaging procedures. According to current official guidelines, the performance of a cerebral CT or MRI-scan is regarded mandatory at this step [52]. The purpose of these morphologic imaging procedures is mainly to exclude non-neurodegenerative causes of cognitive impairment, such as brain tumors or vascular abnormalities. Following the exclusion of other potential causes, the diagnostic assignment to dementia of the Alzheimer type (DAT) or to another neurodegenerative disorder will usually mostly result on the basis of the type of cognitive symptoms.

A definite diagnosis of Alzheimer's disease (AD), however, is currently only possible by post mortem histopathological analysis of the brain. The hallmarks of histological verification of AD are the deposits of ß-amyloid plaques and neurofibrillary tangles [12]. In contrast to other disease entities, the in vivo extraction of tissue for histopathology (e.g. by brain biopsy) can hardly be reasonably justified for diagnosis of dementia. Besides this limitation regarding the definite diagnosis of AD by neuropsychological tools is limited. It is well accepted that the pathology of AD starts years to decades before the onset of cognitive symptoms. Out of this perspective clinical definition of manifest symptomatic dementia will consistently represent an already advanced stage of AD [12,19]. In summary, the early and definite diagnosis of AD is hampered by the low sensitivity and specificity of clinical/neuropsychological evaluation and the limited accessibility of brain tissue for histopathological analysis. On the other hand, considerable progress has recently been made regarding insights into pathomechanisms underlying AD. This progress has been paralleled by increasing efforts to develop new treatment options, mostly directed against amyloid deposition, such as e.g. secretase blockers and immunization strategies [44]. For all causal therapeutic approaches it is of apparent importance to improve early diagnosis of the disease, ahead of the clinical stage of dementia, to allow treatment initiation in low or even asymptomatic stages, i.e. prior to irreversible neuronal damage. Furthermore, the expensive and in part also risky new therapeutic approaches will obviously demand a highly specific diagnosis and tools for monitoring treatment success. As a consequence, the need for a more reliable test for early and specific diagnosis of AD is evident. The most reasonable approach to evaluate the sensitivity of a new diagnostic tool for early diagnosis of AD, is apparently to test its value in AD risk populations. In general, such risk populations can be selected on the basis of different approaches: a) selection of patients with mild cognitive symptoms, not yet sufficient for diagnosis of dementia or b) selection of patients without cognitive symptoms but with a genetic or familiar risk for AD. Regarding a), a diagnostic entity has been created established in the recent years, termed MCI for "mild cognitive impairment". This clinically defined group encloses patients with cognitive symptoms, which do not yet affect their activities of daily living. It has been shown that 15–30% of these patients will develop manifest DAT within a short period of time. Thus, MCI is regarded to define represent a risk population for AD [81]. However, not all MCI-patients will develop DAT and today a definite diagnosis of AD in the stage of MCI is not reliably possible based on neuropsychological tests alone. Regarding b), several known genetic risk factors exist. The Apolipoprotein E (*ApoE*) e4-allele represents the greatest known genetic risk factor for sporadic AD [30]. However, these genetic risk factors do only indicate a theoretical risk and there is no proof for the actual presence of a neurodegenerative disorder. Many carriers of the e4-allele will never develop DAT.

Consequently, the search for specific surrogate markers allowing non-invasive early and specific diagnosis of AD in the stage of MCI or even in asymptomatic stages of the disease has been intensified. Modern imaging procedures hold considerable promise in this context. It has been shown that imaging technologies, such as Magnetic Resonance Imaging (MRI) and Positron Emission Tomography (PET), allow the detection of structural, functional and molecular pathological changes associated with the disease progression and may be superior to neuropsychological testing regarding early and reliable diagnosis of AD [85,118]. As mentioned above, morphological imaging procedures, such as CT and MRI are already an essential part of the standard diagnostic procedure. These techniques can exclude several non-neurodegenerative causes for symtomatic dementia, such as vascular and tumorous diseases or normal pressure hydrocephalus [110]. Furthermore, particularly MRI is capable of precise measurements of regional brain atrophy, e.g. of the hippocampal region. However, morphological changes do show some overlap between different neurodegenerative disorders and the specific proof of a certain neurodegenerative process on the basis of certain patterns of atrophy may be less reliable. In addition, it seems that morphological changes appear later in the progress of disease, subsequently to the onset of neuropathological and functional changes and even to the early symptomatic phase of AD [111]. Thus, morphological structural CT and MRI may not represent the optimal tools for specific differentiation of AD from other types of neurodegeneration so far and particularly not regarding the sensitive early diagnosis of AD in asymptomatic or MCI-stage. Advanced MRI-techniques such as repeated scanning for the longitudinal assessment of changes over time or modern spectroscopy techniques may hold additional options but still need to be evaluated systematically in larger studies.

In contrast to the mentioned morphological structural imaging procedures, functional imaging techniques such as PET allow the sensitive verification of subtle changes in brain physiology. In particular, PET imaging with the glucose analogue [18F]FDG has been demonstrated to visualize even a subtle minor decline of regional neuronal function, which may be one of the very first abnormalities accompanying the onset of AD-pathology in the brain. This imaging tool holds promise to allow a positive proof of AD in very early stages, i.e. prior to major clinical changes and changes in brain structure. However, in current guidelines, functional imaging techniques such as PET are currently still regarded as optional complementary procedures in the diagnostic setup. For several reasons this may have to be reconsidered. First, diagnosis of manifest dementia may have to be replaced by diagnosis of ongoing neurodegeneration in the future. Second, a lot of additional data, confirming the value of the existing procedures such as [18F]FDG PET has been collected. In the current article, the potential value of [18F]FDG, representing one of the most promising and well-established PET techniques, will be discussed with particular regard to early diagnosis of AD in stages ahead of clinically manifest dementia.

## 2. Basics of [18F]FDG PET imaging in AD

Current theories regarding the pathology of AD-development can not be addressed here in detail. In short, AD is today most commonly regarded as a protein aggregation disorder, with extracellular aggregation of ß-amyloid peptide and intracellular aggregation of the tau-protein being the earliest known pathological events of this disease. The ß-amyloid peptides are cleaved from a membrane protein called APP (amyloid precursor protein) and tend to aggregate extracellularly with different affinity, depending on the length the different forms. As a consequence of aggregation, dimers, oligomeres and finally extracellular ß-amyloid plaques are generated. Inside the cell, following hyperphosphorylation the tau-protein, which is usually associated with intracellular microtubuli, aggregates to the so called neurofibrillary tangles. Although the exact mechanism is not yet known, it is assumed that these aggregation pathologies lead to impaired synaptic function, neuronal dysfunction, synaptic loss and finally to neuronal death and resulting brain atrophy [36,99]. Some studies were able to confirm that neuronal loss appears later in the disease progress, when compared to tau and amyloid aggregation pathologies [88]. On the basis of this disease concept, reduced neuronal activity can apparently be regarded as a very early pathology of AD, advancing measurable reductions of brain tissue.

Thus, measuring neuronal dysfunction does obviously represent a promising approach for early detection of AD. With the radiolabeled glucose analogue, [18F]Fluorodeoxyglucose ([18F]FDG), a PET-tracer for in vivo assessment of neuronal function is at hand. The uptake of [18F]FDG parallels the transport of glucose into cells. Subsequently, the tracer is phosphorylized and trapped in the cell and thus allows regional assessment of regional cerebral glucose metabolism (rCGMglc) [86]. It is well known that glucose constitutes the relevant source of energy for the brain and that glucose metabolism is tightly connected to regional neuronal function. Most probably, this coupling is mediated by glial cells. Energy-demanding synaptic activity of a neuron leads to increased glucose uptake into surrounding glial cells, which subsequently transfer lactate as an energy carrier to the neuron [57]. If the [$^{18}$F]FDG PET-examination is performed in resting conditions, the tracer uptake is mainly driven by basal neuronal activity; thus it represents a measure of general neuronal integrity [95]. Inversely, impairment of neuronal function leads to a decrease of regional glucose turnover.

Consequently reductions of cerebral glucose metabolism as a measure of neuronal function in the brain in AD can be expected. In fact, in patients with DAT, characteristic deficits of regional glucose metabolism

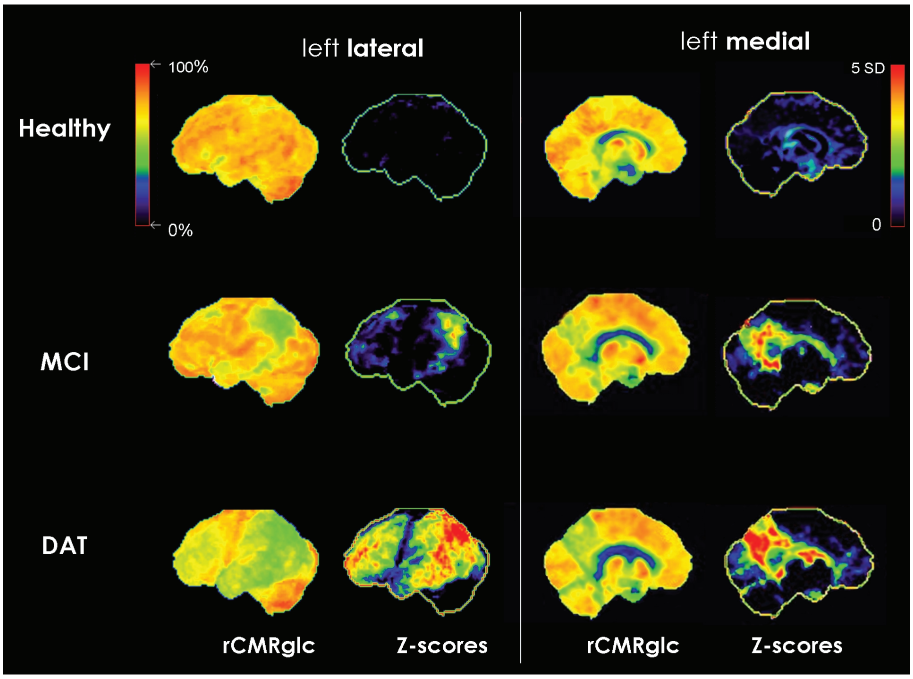

Fig. 1. Surface projections (3DSSP) of typical [18F]FDG PET findings of the left hemisphere in single subjects: healthy control (first row), MCI (second row) and DAT (third row). rCMRglc: glucose metabolism, red/yellow: normally high, green: abnormally low. Z-scores: deviations as compared with a healthy control population, yellow/red: high deviation, blue/black: low/no deviation.

in affected brain regions have often been demonstrated. Typically, temporoparietal cortex, posterior cingulate cortex and frontal cortex show hypometabolic changes. The pattern of affected brain regions fits well to the known deficits in cognitive functions, which have been associated with these particular brain regions, e.g. spatial thinking with the parietal cortex. In contrast, regions such as the sensorimotor cortex and the primary visual cortex are usually spared from hypometabolism, (see Fig. 1) corresponding to the preserved brain functions associated with these regions (vision, sensory and motor function) [38,62,102]. Usually patients show bilateral, but not necessarily completely symmetric hypometabolic areas. According to the disease hypothesis mentioned above, the metabolic deficits in the affected areas of the brain have been mainly assigned to reduced neuronal activity, but also to a number of further mechanisms including impaired metabolic efficiency, or ß-amyloid-induced inhibition of glucose transport [69, 108]. Undoubtedly, disease associated loss of synapses and neuronal death would also contribute to reduced regional metabolic rates in later stages. Thus, there is still some controversy regarding the question whether reduced cerebral glucose metabolism actually represents a cause or a consequence of the disease or both. Either way, the resulting pattern of metabolic changes has a high sensitivity and specificity for AD and, thus, [$^{18}$F]FDG PET does represent a valuable tool for early and also for differential diagnosis of dementia [59,62, 97,102,104].

In general, it has been demonstrated that [18F]FDG-PET imaging offers a tool superior to neuropsychological testing, regarding early diagnosis of AD [85, 102,118]. The true diagnostic quality of neuroimaging procedures can only be evaluated against histopathological assessment. In fact, several studies addressed this issue. Minoshima et al. found that patients with post-mortem histopathological proof of AD showed typical parietotemporal, posterior cingulate, and frontal hypometabolic changes in prior [18F]FDG-PET scans [64]. Also Hoffmann et al reported that parietotemporal hypometabolism is the typical abnormality in patients with verified AD [42]. In a large multicenter trial Silverman et al were able to demonstrate a sensitivity of 94% and a specificity of 73% of a diagnostic setup including [18F]FDG PET regarding the diagnosis of AD, as verified by histopathological proof of disease [102]. In contrast, in studies using histopathology as the goldstandard for neuropsychological evaluation, distinctly lower values of sensitivity (85%) and specificity (55%) have been found [42, 56]. These studies provide convincing evidence that diagnostic setups including [18F]FDG PET show higher accuracy than neuropsychological evaluation alone regarding diagnosis of true AD.

In most of the mentioned PET-studies, the sensitivity of [18F]FDG PET for detection of AD has been found to be very high (>90%), whereas less high values have usually been reported for specificity. However, a number of studies demonstrated that automated procedures for observer-independent image evaluation may lead to a considerable improvement of this issue. A common approach consists in automated interindividual 3D-stereotactic spatial normalization of the image data, allowing voxel-based statistical comparison of patients data with data from healthy control subjects. Several programs for this type of analysis are available (e.g. Neurostat 3D-SSP, University of Michigan, USA or SPM, Wellcome Dept. London, GB). These programs facilitate the assessment of statistically significant abnormalities and potentially even the automated assignment to a diagnostic category. Their value in the diagnosis of AD has been extensively tested [8, 26,28,39,41,65]. A large multicenter study ("NEST-DD") was able to prove that the application of this type of voxel-based automated data-analyses to [18F]FDG PET data leads to an improved specificity of 93%, while maintaining a sensitivity of 93%, regarding the diagnosis of AD [39,41]. With this approach, even in very mild dementia (at MMSE 24 or higher) sensitivity still reached 84% and specificity 93%.

Another option for objective semiquantitative analysis of [18F]FDG PET data may be found in the application of regional ROI's. For this kind of approach, the patient's PET and MRI image data have often been coregistered at first in order to define anatomical ROI's on individual MRI data, which have been applied to the corresponding PET data, subsequently [22]. This type of approach, however, is time-consuming and requires manual ROI-definition. Data-analysis procedures allowing automated ROI-definition may be less susceptible for observer-dependent variations. Mosconi et al. introduced an automated and anatomically valid mask technique to sample the hippocampus on PET data (so-called HipMask). They were able to document hippocampal hypometabolism in DAT with this technique and to demonstrate that it may improve discrimination between healthy subjects, MCI and DAT-patients [69].

In summary, there is good evidence that diagnostic algorithms including [18F]FDG PET achieve better results than neuropsychological testing alone. It has been shown that [18F]FDG PET may actually be included into algorithms of dementia diagnostics in a cost-efficient way and that it indeed leads to an improvement of patient management, including therapy and care [75, 100,101]. So far, comparable results for most other imaging procedures are still pending. Of the existing MRI-techniques, MRI-based volume measurements of atrophy have been most detailed evaluated regarding the diagnostic value in detection of Alzheimer's disease [50,55]. Particularly, volumetry of the hippocampus appears to be able to differentiate between AD and healthy volunteers and some studies also document a correlation between in vivo MR-volumetry and post mortem neuropathological classification of AD [49]. However, the utility of these volumetric procedures to reliably classify the individual patient in a clinical setting has not been sufficiently verified and considerable scepticism about the added value of these procedures to standard clinical evaluation still remains [24,31,48,94, 113]. Alternative promising MR-imaging procedures, which may offer insights into various parameters of AD-pathology in the brain are also still in the process of validation [50].

So far, the number of studies directly comparing the accuracy of MRI-volumetry and [18F]FDG PET for diagnosis of AD is very limited. A small number of studies, however, indicate that [18F]FDG PET yields a better diagnostic performance for the discrimination between AD patients and healthy control subjects than does the morphologic assessment of atrophy, e.g. by voxel-based morphometry (VBM) [58]. Also a study by Kawachi and colleagues showed a slightly higher accuracy for detection of AD for [18F]FDG PET compared to VBM-MRI [51]. This impression is further confirmed by a number of studies demonstrating that the hypometabolic changes in measured by PET in AD exceed structural changes and are not representing an artefact due to an increase in CSF space induced by atrophy, but reflect a true metabolic reduction per gram of tissue [11,46]. Studies, which applied partial volume effect correction to eliminate the effects of atrophy on PET signal, consistently demonstrated that FDG uptake is reduced in AD regardless of whether or not partial volume effect correction is applied, supporting the notion that the reduced FDG uptake in these areas is not the result of atrophy. In a recent direct voxel-based comparison between gray matter hypometabolism and atrophy in Alzheimer's disease, Chetelat et al. were able to demonstrate that hypometabolism significantly exceeds atrophy in most altered structures, particularly in the posterior cingulate-precuneus, orbitofrontal, inferior temporo-parietal, parahippocampal, angular and fusiform areas in AD [17]. All these studies further support the theory that functional changes advance major atrophy and, in consequence, functional imaging may be more sensitive to detect AD compared to structural imaging.

## 3. [18F]FDG PET in MCI

As mentioned above, following a period free of symptoms, it is assumed that patients go through a stage of so called mild cognitive impairment (MCI) for 5–10 years (often particularly in the memory domain), before clinically manifest dementia can be diagnosed [81–83]. Clinical diagnosis of MCI is defined by a loss of cognitive functions, which exceeds common age-associated changes but does not yet allow for the diagnosis of dementia (i.e. patients are not impaired regarding their activities of daily living) [83]. Thus, the MCI group is regarded as a risk population for AD, and may be of particular interest regarding new treatment trials [34]. Consequently, also the current guidelines of the American Academy of Neurology recommend that patients with Mild Cognitive Impairment should be identified and monitored for progression to AD, which is regarded to be a likely event [52].

So far, a number of studies have evaluated the value of [$^{18}$F]FDG PET in the diagnostic assessment of MCI. In several cross-sectional studies (some of them large multicenter studies with >100 MCI subjects), it has been consistently demonstrated that [$^{18}$F]FDG PET imaging can reliably differentiate groups of MCI-patients from healthy controls and on the basis of specific hypometabolic patterns [23,26,27,66,74,76,78]. The most consistent abnormal finding, described in the majority of these studies is the hypometabolism in the posterior cingulate cortex. Depending on the study, this finding has been accompanied by more or less involvement of additional cortical regions affected by hypometabolism in manifest AD-dementia, such as the temporoparietal cortex and frontal cortical regions. As in AD occipital and sensorymotor cortical areas are spared from hypometabolic changes consistently.

There has been some controversy regarding the hypometabolic involvement of the medial temporal cortex, particularly of the hippocampus. Whereas some studies show hyometabolism in this region in MCI [20, 22,73], others do not show major medial temporal hypometabolism in MCI or AD-dementia [6,27,40,63,66, 98]. The medial temporal cortex and in particular the hippocampus has often been discussed as a region in the brain early affected by pathological changes including atrophy in the course of AD. Given the fact that a major role in memory function has often been assigned to this brain structure, it seems reasonable to look for hypometabolism in this region in MCI. The fact that data on hypometabolic changes in MCI in this region are thereto limited may have two apparent reasons: first the assessment of cerebral hypometabolism in this region may be aggravated by the limited spatial resolution of PET. In fact, studies showing hypometabolism in the hippocampus predominantly applied specific data processing technologies, such as MR-based hippocampal ROI-definition or voxel-based analysis following correction of regional partial volume effects [61,69]. Thus, in other studies employing less specific approaches for the assessment of this region, this abnormality may have been missed. Another potential reason for the lack of hypometabolic findings in the hippocampus may be found in a regional compensatory effect, which has been increasingly discussed recently. The basic idea underlying this hypothesis is that a loss in the number of neurons in the hippocampus will be compensated by increased activity of the remaining neurons. Several studies support this view [17,47,98]. Samuraki et al. found reduced [18F]FDG uptake in posterior cingulate and parietal temporal lobe in mild AD but relatively preserved uptake in the MTL, including hippocampal areas, despite gray matter loss. These findings persisted even after partial volume correction. The authors suggested that compensatory mechanisms in the hippocampus may be responsible for these findings [98]. Similarly, in a recent study Chetelat et al. demonstrated that hypometabolism in the brain exceeds atrophy in several regions including the posterior cingulate, whereas less hypometabolism was found in the hippocampus compared to distinct atrophy in this region. Again, the authors suggested that compensatory mechanisms may be responsible for these findings [17].

Finally, some of the variance in the metabolic patterns detected in the MCI-group may be attributed to the type of criteria used for definition of MCI (amnestic, multiple domain versus single domain, etc.), to neuropsychological profiles [43,74], as well as to the well-known heterogeneity of the MCI-group itself. Some MCI-subjects are suffering from diseases other than AD, some will convert to dementia later than others and some may even get better over time and never develop dementia [82]. Thus, the most important diagnostic challenge is not to distinguish MCI-patients as a group from healthy controls, but rather the identification of those individual patients who will deteriorate to manifest dementia over time (i.e. those subject who

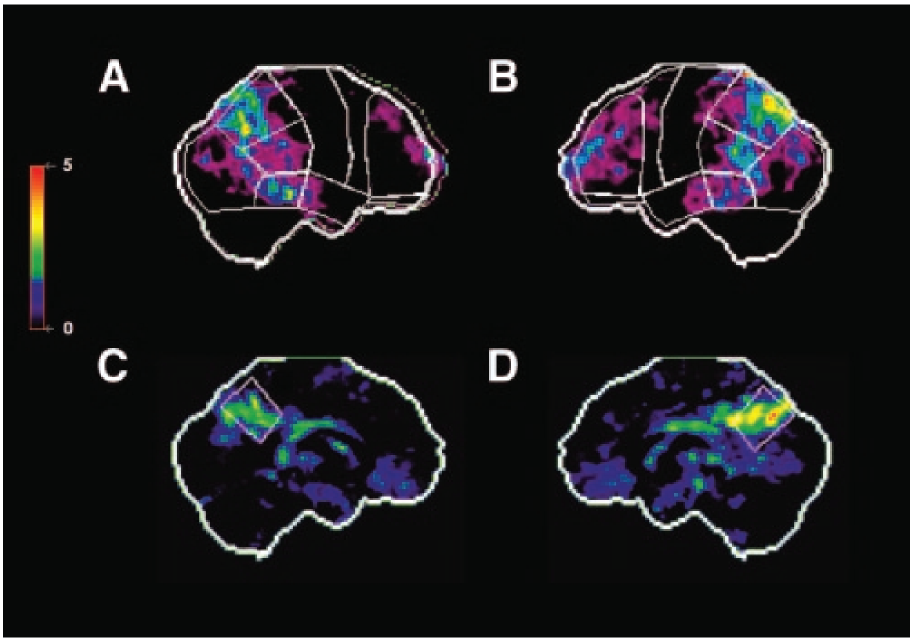

Fig. 2. Baseline [18F]FDG PET findings in a single MCI patient, who showed clinical progression to DAT within 16 months. Surface projections of statistical abnormalities (z-scores) as compared with a healthy control population are displayed. Predefined anatomic surface ROIs are depicted as white lines. Significant hypometabolism in bilateral temporoparietal and frontal cortex and in the posterior cingulate cortex is apparent. (A) Right lateral. (B) Left lateral. (C) Left medial. (D) Right medial. Reproduced with permission from Drzezga et al 2005 (Drzezga et al. 2005).

are actually suffering from AD). As discussed above, clinical/neuropsychological examination does not consistently permit a reliable prediction of conversion to dementia in the group of MCI-patients [5,83]. Thus a number of studies have been carried out to identify the predictive value of [18F]FDG PET as a biomarker of ongoing AD in MCI [6,16,26,27,40,45,71,78,102]. Numbers of included patients in these studies ranged beween 17 and 55, mean follow-up periods ranged between 1–4 years and observed conversion rates ranged between 22–45%. All of these studies were able to identify typical hypometabolic changes in [18F]FDG PET baseline examinations of MCI-patients, associated with later conversion to manifest AD-dementia, whereas stable subjects showed less or no abnormalities. Again, most consistently across those studies, posterior cingulate hypometabolism was identified as a key finding, predicting later conversion of MCI to AD, followed by parietotemporal and frontal hypometabolism. Generally high sensitivity and specificity values were calculated (75%–100%) for [18F]FDG PET. The ultimate value of a diagnostic biomarker for predicting the clinical course can only be defined by comparison of baseline rating values against results from longitudinal follow up in the individual patient. A small number of studies tried to follow this longitudinal approach in MCI. Silverman et al included a subset of 55 patients with only mild cognitive impairment in their longitudinal multi-center study. They were able to demonstrate that progression of cognitive deterioration was predictable on the basis of baseline PET-findings with a sensitivity of 91% and 75% specificity in this sample [102]. A smaller study employing automated image analysis and classification of patients according to predefined criteria at baseline was able to achieve a sensitivity of 92% and a specificity of 89% (positive predictive value 85% negative predictive value 94%) regarding conversion to AD 16 months later (see Fig. 2) [26].

In MCI, correlation of imaging data with post mortem data has not been frequently performed. Two studies demonstrate that [18F]FDG has a high sensitivity regarding identification of actual ongoing AD in the stage of MCI or even in cognitively healthy subjects, as verified by post mortem assessment years after the original PET examination [70,102]. Mosconi et al did also demonstrate that PET based diagnosis remained consistent in repeated measurements in the same subjects over longer time periods. Silverman and colleagues demonstrated in their multi-center study that [18F]FDG PET was able to detect ongoing Alzheimer's disease in subjects with mild cognitive symptoms as verified later by post mortem neuropathological assessment with 95% sensitivity and 71% specificity [102].

Again, as for DAT, it has been shown also for MCI that automated data-analysis procedures minimizing observer-influence may considerably improve image assessment. Particularly the automated individual comparison of patient data with healthy control populations shows promising results [27,28,61,66,74]. In a recent study it has been demonstrated that the careful selection of a longitudinally controlled truly healthy control population may be crucial for the quality of the results gained from this type of study [74]. Furthermore, it has been shown that the selection of the region used for normalization of the individual data may influence the sensitivity of the examination [115].

Regarding this array of promising results, it is not surprising that several studies were able to demonstrate higher accuracy of [18F]FDG for prediction of AD-dementia in MCI-patients, compared to neuropsychological examination [71,102]. Even when post-mortem neuropathological evaluation was considered the gold-standard, it was obvious that adding [18F]FDG to the diagnostic setup did improve prediction accuracy [102]. In contrast to these findings, there is considerably more controversy regarding the added value of volumetric MRI examinations regarding early identification of AD. Currently, volumetric assessment of the medial temporal lobe/hippocampus can be regarded the best evaluated parameter regarding the identification of AD in early stages of the disease [4,18,24,29,54,109,112]. However, the number of studies reporting on the actual predictive value of these measurements in longitudinal studies is relatively limited and the actually reported predictive values are rather low. E.g. Korf et al reported a sensitivity of 70% and a specificity of 68% (positive predictive value 68%, negative predictive value 70%) for visually assessed medial temporal lobe atrophy [54]. Devanand demonstrated sensitivity and specificity of 67%: and 80%, respectively for measurements of hippocampus and entorhinal cortex [24]. They conclude that the added value of MR imaging to clinical parameters such as age and cognitive variables remains rather limited, as far as prediction of clinical course is concerned. Also Fleisher et al. in their recent study summarize that in moderate stages of amnestic MCI, common cognitive tests provide better predictive accuracy than MRI measures of whole brain, ventricular, entorhinal cortex, or hippocampal volumes for assessing progression to DAT [31].

Some studies imply that longitudinal assessment of atrophy rates in the hippocampus and possibly also even of the whole brain may be more sensitive regarding future cognitive deterioration, and thus may have predictive value in early stages such as MCI [103, 106]. However, e.g. Jack et al. discussed in their study that the considerable overlap between converters and non-converters indicates that these measures are unlikely to provide absolute prognostic information for individual patients [48]. Furthermore concerns have been raised regarding the clinical practicability for serial MRI imaging regarding increased costs, complexity of serial image coregistration and the clinical need to promptly establish the diagnosis [24]. It may also be mentioned in this context that a recent post mortem study correlating post mortem MRI-measurements of medial temporal lobe atrophy (MTA) with specific neuropathology demonstrated that MTA is neither specific nor does it show a very high sensitivity (63%) for AD [7].

In correspondence to these findings and interpretations, studies on direct comparison of the diagnostic value of [18F]FDG PET and MRI-methods in MCI predominantly report relatively higher accuracy of PET. As an example, De Santi et al. demonstrated that [18F]FDG PET measurements achieved higher accuracy compared to MRI volume measurements regarding diagnostic classification of MCI [22]. Also Yuan et al. recently demonstrated that [18F]FDG PET can be regarded superior to MRI in predicting conversion in MCI [116]. These findings correspond well to the hypothesis that functional changes appear earlier in the history of AD and that hypometabolism advances and exceeds atrophy and is better suited for early diagnosis [58,72,98].

Apart from volumetric assessment of regional brain atrophy, various other modern MRI-techniques may allow to assess different aspects of Alzheimer's disease pathology, such as diffusion weighted imaging, perfusion imaging and arterial spin labelling, assessment of resting state activity and spectroscopy. Most of these techniques, however, have only been studied in small samples and information on their value for early diagnosis of AD is yet limited. Their predictive value regarding conversion from MCI to AD in large cohorts remains to be established [24,50].

Consequently, the recent guidelines (2001, reaffirmed 2004) for dementia diagnosis of the American Academy of Neurology (AAN) explicitly state that linear or volumetric MR or CT measurement strategies should not be included in the routine evaluation of the demented patient. It is concluded that measurement of hippocampal atrophy by MRI may not be useful in clinical practice because of its low precision. It is also discussed that [18F]FDG-PET appears superi-

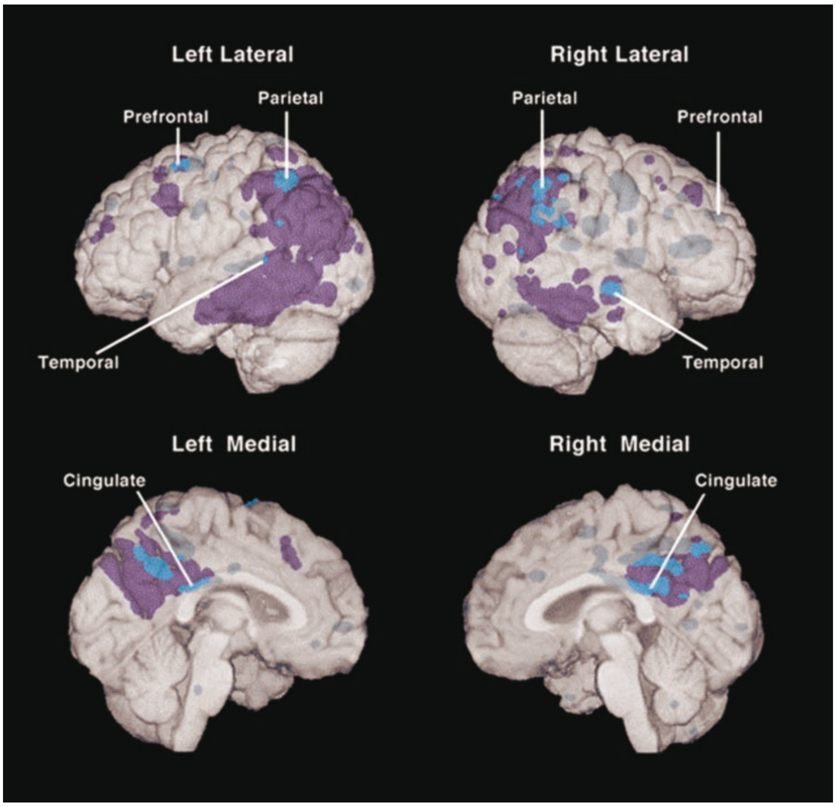

Fig. 3. Regions of the brain with abnormally low CMRgl in young adult carriers of the APOE e4-allele and their relation to brain regions with abnormally low CMRgl in patients with probable DAT. The dark (purple in coloured online version) are regions in which CMRgl was abnormally low only in the patients with DAT, the bright (bright blue in the coloured online version) areas are regions in which CMRgl was abnormally low in both the young adult e4 carriers and patients with probable DAT, and the grey areas (muted blue in the coloured online version) are regions in which CMRgl was abnormally low only in the e4 carriers. (Lines point to the locations of the e4 carriers' most significant CMRgl reductions. Reproduced with permission from Reiman et al. 2004 (Reiman et al. 2004)© 2003 by The National Academy of Sciences of the USA.

or to MRI measures of hippocampal atrophy because changes in cerebral glucose metabolism antedate the onset of memory decline, whereas the MRI hippocampal changes do not. Thus, it is concluded in these guidelines that PET scanning appears to have promise for use as an adjunct to clinical diagnosis, but further prospective studies with PET are needed to establish its added value over a competent clinical diagnosis [52]. Interestingly in these guidelines the evaluation and clinical monitoring of persons with MCI is explicitly recommended, due to their increased risk for developing dementia [29,53]. However, no detailed discussion on the value of imaging biomarkers in the stage of MCI is provided. These guidelines date back to 2001 and since then abundant literature has been added, particularly on the value [18F]FDG PET for early diagnosis of AD in MCI. Thus, it may be considered to include this aspect in upcoming versions of these guidelines.

When using [18F]FDG PET for diagnosis in MCI and AD, it needs to be kept in mind that the degree of cognitive impairment will not necessarily correlate linearly with the extent of cerebral hypometabolism in each subject. A number of studies were able to demonstrate that subjects who were expected to have higher cognitive reserve, i.e. due to higher levels of education, showed more pronounced hypometabolism as compared to subjects with lower levels of education and similar degree of cognitive impairment [80,107]. Recently, a study was able to demonstrate that this type of cognitive reserve effects may also be observed in MCI subjects [35]. These cognitive reserve effects further underline that a diagnosis based on cognitive symptoms alone may not be suitable to assess the extent of damage in the brain.

## 4. [18F]FDG PET in asymptomatic risk persons

There is common agreement that the brain pathology of AD starts years before onset of even mild clinical symptoms, i.e. even ahead of the stage of MCI. In principle, it may make sense to start new therapeutic strategies even in presymptomatic stages, to avoid neuronal damage before it leads to cognitive impairment. Thus, the identification of biomarkers allowing the diagnosis of AD in asymptomatic stages appears to be a worthwhile aim. According to current diagnostic guidelines, there are still insufficient data to make any recommendations regarding cognitive screening of asymptomatic individuals [82]. Thus, in a first step it appears reasonable to focus on healthy subjects, who bear an increased risk for developing AD-type dementia later in life, i.e. they potentially may suffer already from ongoing neuropathology, even without apparent cognitive symptoms. Apparently, subjects carrying genes encoding for the familiar forms of Alzheimer's diseases represent such a risk population. In fact one study was able to demonstrate hypometabolic abnormalities in subjects carrying mutations in the Presenilin 1 gene, responsible for early-onset autosomal dominant AD (FAD) [72]. Presymptomatic FAD individuals showed hypometabolic changes in [18F]FDG PET scans, consistent with the typical AD PET pattern in the relative absence of structural brain atrophy. The authors concluded that these data further suggest that [18F]FDG PET measures may serve as biomarkers for the preclinical diagnosis of AD. However, the familiar cases of autosomal dominant inherited AD only represent a small percentage of all AD-cases and they are different regarding onset and clinical course compared to the frequent sporadic form. Thus, findings obtained in this population may not be easily transferred to the general population of sporadic AD. Apart from autosomal dominant forms of AD, a higher risk for sporadic AD may be defined by familiar disposition (positive familiy history for AD), or by known factors of genetic susceptibility such as the *ApoE* e4-positive genotype. The *ApoE* e4-allele represents the greatest known risk factor for sporadic AD, increasing the relative risk to suffer from AD for about 3–15 times [33], depending on the e4-allele frequency. Correspondingly, several studies have been able to demonstrate hypometabolic abnormalities in healthy carriers of the *ApoE* e4-genotype compared to e4-negative controls. For example, Reiman et al showed reduced glucose metabolism in e4-positive subjects in the same regions of the brain as in patients with probable Alzheimer's disease (posterior cingulate, parietal, temporal, and prefrontal regions) [91]. Members of the same group later demonstrated that even relatively young (20–39 years) e4-carriers had abnormally low rates of glucose metabolism bilaterally in the posterior cingulate, parietal, temporal, and prefrontal cortex and that the e4-gene dose is correlated with lower glucose metabolism in each of these brain regions (see Fig. 3) [92,93]. Furthermore in several studies specific decline of glucose metabolism over time in the AD-typical regions has been demonstrated in cognitively healthy e4-carriers [90,105]. Correspondingly, more pronounced hypometabolism was detected in e4-positive subjects with manifest DAT compared to age-matched e4-negative DAT-patients [31]. Conclusively, it has been suggested that brain imaging techniques could be used to evaluate putative AD prevention therapies in cognitively normal *ApoE* epsilon4 carriers and that they could help guide the investigation of a molecular mechanism associated with AD vulnerability [89]. Apart from subjects with *ApoE* e4-positive genotype, another very recent study was also able to demonstrate similar hypometabolic changes in subjects with maternal history of AD, who are also at higher risk for dementia [70].

However, both family history and the *ApoE* genotype do only indicate a theoretical risk and are no proof for the actual presence of a neurodegenerative disorder. Many carriers of the 4-allele will never develop DAT. This implies that significant abnormalities were detected in the mentioned studies in the risk populations when compared with healthy controls, although the risk-groups will most probably have contained several subjects who were not suffering from ongoing neurodegeneration. Thus, it appears particularly interesting to evaluate the value of these detected abnormalities, regarding the prediction of cognitive decline in individual subjects in the future. In a single [18F]FDG PET study De Leon et al. were able to demonstrate that baseline metabolic reductions in the entorhinal cortex of healthy subjects accurately predicted the conversion from normal to MCI within a 3-year follow up period [23]. In the subjects who declined, the baseline entorhinal metabolism predicted longitudinal memory and temporal neocortical metabolic reductions. More data needs to be collected in larger longitudinal studies over long time intervals, to evaluate the utility of [18F]FDG PET for individual prognosis/early detection of AD in healthy subjects and its actual value as a screening tool.

## 5. Multi-modal approaches

It is commonly assumed that AD represents a complex multifactorial disorder, which will not necessarily follow the identical course in every single patient. Thus, in general the most appropriate way to ensure reliable early diagnosis of AD may be found in the combination of several diagnostic tools, focussing on different aspects of the disease. This includes clinical and neuropsychological testing as well as different types of biomarkers such as the assessment of genotype, cerebrospinal fluid markers and functional/molecular and structural imaging procedures. A number of studies suggest superior performance of diagnostic setups combining different diagnostic markers for detection of AD in the stage of MCI. This includes studies on the combination of imaging and non-imaging biomarkers [24, 29,68,71,105,111], as well as on the combination of different imaging modalities [27,51,114]. Two studies were able to demonstrate that the combination of the *ApoE*-genotype, encoding for AD-susceptibility, with [18F]FDG-PET as a marker of actual brain pathology, could significantly improve either sensitivity or specificity of DAT-prediction in MCI-patients [29,71]. Also the current AAN guidelines suggest that combining medial temporal measures with other markers, such as functional neuroimaging or *ApoE*-genotyping, may improve diagnostic accuracy [52].

The discussion about combining different diagnostic entities to find to a comprehensive diagnosis may gain even more momentum in the future, regarding two recent developments. One being new PET tracers for molecular imaging of amyloid plaque deposition such as [11C]PIB [28,79,96]. First studies suggest a high predictive value of this type of tracer for prediction of AD in MCI [35] and a small study suggests superior classification of AD compared to [18F]FDG-PET [77]. However, the relatively high numbers of PIB-positives in older asymptomatic subjects require further longitudinal studies [67]. [18F]FDG-PET may be highly valuable to detect the onset of actual functional pathology in PIB-positive subjects. Thus, PIB-PET may serve as a risk-marker, whereas FDG-PET may denote the actual onset of disease. A second important development lies in the current efforts to establish a new hybrid instrument, combining PET and MR imaging technology into one single scanner. This hybrid imaging technology may significantly influence diagnostic protocols, as it will allow simultaneous acquisition of functional, molecular and structural information in one session in the future [17,18].

## 6. Conclusion

In conclusion, imaging of the cerebral glucose metabolism with [18F]FDG-PET as a marker of neuronal function can be regarded as a highly sensitive and specific tool for early and accurate diagnosis of Alzheimer's disease. It has been demonstrated even by post mortem neuropathological assessment that this imaging technique shows better diagnostic performance than exclusive neuropsychological evaluation. Furthermore excellent performance of [18F]FDG for early diagnosis of this disorder even in early mild symptomatic stages (MCI) has been demonstrated, i.e. before a diagnosis of dementia is possible by neuropsychological assessment. The documentation of abnormal findings in risk persons even in asymptomatic stages underlines the very high sensitivity of this tool. Current data indicate that [18F]FDG PET shows superior performance regarding prediction of cognitive decline in MCI, compared to established structural imaging methods, such as MR-volumetry. These findings are corresponding to the hypothesis that functional changes occur prior to structural changes in the brain. Current guidelines on dementia diagnostics discuss a potential role of [18F]FDG imaging for diagnosis of dementia and discuss its superiority compared to MR-volumetry, but they do not explicitly recommend the performance of [18F]FDG PET. However, the role of imaging procedures for diagnosis of AD in stages ahead of manifest dementia such as MCI is not discussed in these guidelines, although careful diagnostic evaluation of MCI-patients, representing a high-risk group for dementia, is strongly advised. Regarding the fact that AD-pathology starts years to decades prior to the onset of clinically manifest dementia, it becomes obvious that diagnostic efforts should be directed towards diagnosis of Alzheimer's "Disease" rather than of Alzheimer's "Dementia". Neuronal dysfunction, which can be detected with [18F]FDG PET may be regarded as the actual onset of Alzheimer's disease in the brain, disregarding symptomatic stage. In this context and regarding the abundant amount of new information on the value of [18F]FDG PET, which has been collected in the recent years, it may be considered to include [18F]FDG -PET as a highly sensitive marker of disease in early stages in the current guidelines for diagnosis of "dementia". Apart from the value of [18F]FDG PET alone, certain combinations of this imaging marker with other biomarkers such as genotype or CSF-markers may offer promising approaches to assess individual prognosis in AD-patients. New developments in the field of imaging, such as tracers for amyloid plaque imaging, modern MR-procedures and new hybrid imaging technologies such as PET-MR may add further material to this discussion. In the future, these exciting options may offer the opportunity to address a number of different diagnostic questions, e.g.: a) the detection of protein deposition pathology with amyloid plaque imaging, b) the onset of neuronal dysfunction (i.e. the beginning of actual Alzheimer's disease) with [18F]FDG and c) the presence of brain atrophy and other structural and biochemical consequences of the disease with MRI.

It can be summarized that the value of [18F]FDG for early diagnosis of AD has been systematically proven. At present, comparable qualities regarding sensitivity, specificity, value for therapy control and even cost-efficiency in AD have not been documented for other imaging procedures. Therefore, PET can be regarded as a method of choice regarding diagnosis of ongoing AD-neurodegeneration, particularly in patients with mild cognitive impairment.

## Acknowledgment

This work has been supported in part by DFG-grants (Deutsche Forschungsgemeinschaft) Project Numbers: DR 445/3-1, DR 445/4-1 (Drzezga). The author thanks Nicola Karina for the careful review of the manuscript.
